# The Reactome Pathway Knowledgebase 2024

**DOI:** 10.1093/nar/gkad1025

**Published:** 2023-11-06

**Authors:** Marija Milacic, Deidre Beavers, Patrick Conley, Chuqiao Gong, Marc Gillespie, Johannes Griss, Robin Haw, Bijay Jassal, Lisa Matthews, Bruce May, Robert Petryszak, Eliot Ragueneau, Karen Rothfels, Cristoffer Sevilla, Veronica Shamovsky, Ralf Stephan, Krishna Tiwari, Thawfeek Varusai, Joel Weiser, Adam Wright, Guanming Wu, Lincoln Stein, Henning Hermjakob, Peter D’Eustachio

**Affiliations:** Ontario Institute for Cancer Research, Toronto, ON M5G0A3, Canada; Oregon Health and Science University, Portland, OR 97239, USA; Oregon Health and Science University, Portland, OR 97239, USA; European Molecular Biology Laboratory, European Bioinformatics Institute (EMBL-EBI), Wellcome Genome Campus, Hinxton, Cambridgeshire CB10 1SD, UK; Ontario Institute for Cancer Research, Toronto, ON M5G0A3, Canada; College of Pharmacy and Health Sciences, St. John's University, Queens, NY 11439, USA; European Molecular Biology Laboratory, European Bioinformatics Institute (EMBL-EBI), Wellcome Genome Campus, Hinxton, Cambridgeshire CB10 1SD, UK; Department of Dermatology, Medical University of Vienna, 1090 Vienna, Austria; Ontario Institute for Cancer Research, Toronto, ON M5G0A3, Canada; Ontario Institute for Cancer Research, Toronto, ON M5G0A3, Canada; NYU Grossman School of Medicine, New York, NY 10016, USA; Ontario Institute for Cancer Research, Toronto, ON M5G0A3, Canada; Oregon Health and Science University, Portland, OR 97239, USA; European Molecular Biology Laboratory, European Bioinformatics Institute (EMBL-EBI), Wellcome Genome Campus, Hinxton, Cambridgeshire CB10 1SD, UK; Ontario Institute for Cancer Research, Toronto, ON M5G0A3, Canada; European Molecular Biology Laboratory, European Bioinformatics Institute (EMBL-EBI), Wellcome Genome Campus, Hinxton, Cambridgeshire CB10 1SD, UK; NYU Grossman School of Medicine, New York, NY 10016, USA; Ontario Institute for Cancer Research, Toronto, ON M5G0A3, Canada; Institute for Globally Distributed Open Research and Education (IGDORE); European Molecular Biology Laboratory, European Bioinformatics Institute (EMBL-EBI), Wellcome Genome Campus, Hinxton, Cambridgeshire CB10 1SD, UK; European Molecular Biology Laboratory, European Bioinformatics Institute (EMBL-EBI), Wellcome Genome Campus, Hinxton, Cambridgeshire CB10 1SD, UK; Open Targets, Wellcome Genome Campus, Hinxton, Cambridgeshire CB10 1SD, UK; Ontario Institute for Cancer Research, Toronto, ON M5G0A3, Canada; Ontario Institute for Cancer Research, Toronto, ON M5G0A3, Canada; Oregon Health and Science University, Portland, OR 97239, USA; Ontario Institute for Cancer Research, Toronto, ON M5G0A3, Canada; Department of Molecular Genetics, University of Toronto, Toronto, ON M5S 1A1, Canada; European Molecular Biology Laboratory, European Bioinformatics Institute (EMBL-EBI), Wellcome Genome Campus, Hinxton, Cambridgeshire CB10 1SD, UK; NYU Grossman School of Medicine, New York, NY 10016, USA

## Abstract

The Reactome Knowledgebase (https://reactome.org), an Elixir and GCBR core biological data resource, provides manually curated molecular details of a broad range of normal and disease-related biological processes. Processes are annotated as an ordered network of molecular transformations in a single consistent data model. Reactome thus functions both as a digital archive of manually curated human biological processes and as a tool for discovering functional relationships in data such as gene expression profiles or somatic mutation catalogs from tumor cells. Here we review progress towards annotation of the entire human proteome, targeted annotation of disease-causing genetic variants of proteins and of small-molecule drugs in a pathway context, and towards supporting explicit annotation of cell- and tissue-specific pathways. Finally, we briefly discuss issues involved in making Reactome more fully interoperable with other related resources such as the Gene Ontology and maintaining the resulting community resource network.

## Graphical Abstract

**Graphical Abstract:**
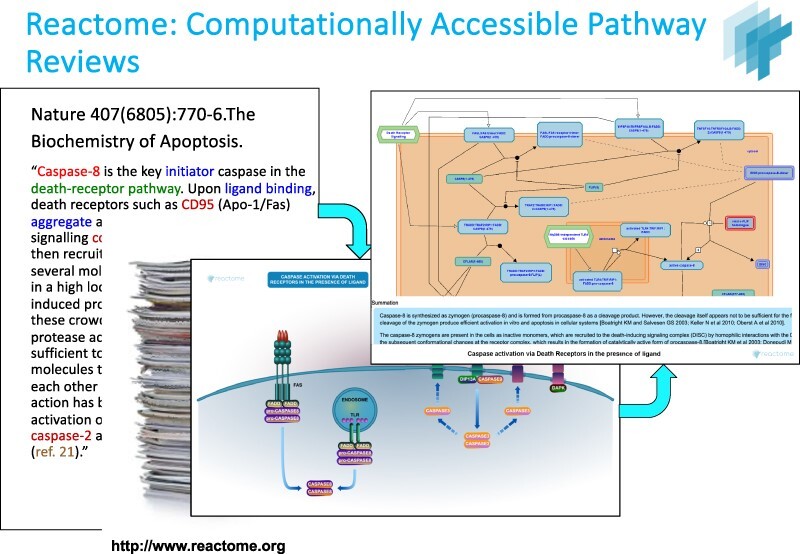

## Introduction

At the cellular level, biological processes of cells and tissues can be represented by networks of molecular reactions that enable signal transduction, transport, DNA replication, protein synthesis, and intermediary metabolism. Various online resources capture this information at the level of individual reactions, such as Rhea (1), or at the level of reaction sequences covering many domains of biology, such as KEGG (2) or MetaCyc (3). The Reactome Knowledgebase is distinctive in focusing its manual annotation effort on a single species, *Homo sapiens*, and applying a single consistent data model across all domains of biology. Processes are systematically described in molecular detail to generate an ordered network of molecular transformations, resulting in an extended version of a classic metabolic map (4) generally compliant with the SBGN process description standard (5). The Reactome Knowledgebase systematically links human proteins to their molecular functions, providing a resource that is both a textbook of biological processes and a tool for discovering novel functional relationships in data such as tissue-, cell- or physiological state-specific gene expression, catalogs of somatic mutations in tumor cells, or likely effects of drugs based on their known interactions with proteins in annotated pathways.

Reactome (version 86—September 2023) has entries for 11 148 protein-coding genes involved in 14 803 reactions annotated from 37 156 literature references (Table 1). These reactions are grouped into 2647 pathways (e.g. Interleukin-15 signaling) collected under 29 superpathways (e.g. Immune System) that describe normal cellular functions. A Disease superpathway includes pathways driven by germline and somatic mutations, and ones due to the actions of genes of infectious bacteria and viruses. Genetic disease annotations cover 4919 variant proteins and post-translationally modified forms of them, derived from 354 human gene products, and annotate 1544 disease-specific reactions tagged with 623 Disease Ontology terms (6). Infectious disease pathways include the effects of bacterial toxins, aspects of infection by Leishmania, Listeria and Mycobacteria, and viral infection mediated by influenza, HIV, human cytomegalovirus, and SARS-CoV-1 and -2. In addition, Reactome describes the modulating effects of 1119 drugs on both normal and disease processes.

**Table 1.** Reactome content, version 78 (9/2021) versus 86 (9/2023)

Data type	Release 78	Release 86	Change
Human proteins	10 726	11 148	422
Proteoforms	29 466	30 338	872
Chemicals	1940	2025	85
Reactions	13 890	14 803	913
Human disease proteins	352	354	2
Disease variants	4603	4919	316
Chemical drugs	468	1033	565
Protein drugs	39	86	47
Literature references	34 025	37 156	3131

‘Human proteins’ is the number of human UniProt entries (not counting isoforms) annotated in Reactome. Each may be represented as multiple proteoforms to account for covalent modifications and subcellular locations. ‘Disease proteins’ are ones whose germline or somatic variation gives rise to proteins with altered, pathogenic functions. ‘Disease variants’ is the total number of such variant alleles annotated in Reactome.

## Annotating the whole human proteome

The goal of the Reactome project is to describe the molecular function of every human protein in the context of a reaction network (7,8). The fraction of the human proteome annotated in Reactome is thus the core metric of progress; other measures are driven by work towards this goal. The 11148 protein gene products now annotated in Reactome are 56.2% of the 19 831 protein-coding genes predicted in the current (GRCh38.p14) human genome assembly (https://ensembl.org/Homo_sapiens/Info/Annotation). A recent survey (9) suggests that experimental evidence is available for ∼68% of these predicted gene products, or 13 500, leaving ∼2350 annotatable human proteins not yet in Reactome and ∼6300 ‘dark’ proteins (10) whose functions are not yet directly accessible for annotation.

Placing proteins with unknown functions into the context of pathways using evidence from high-throughput surveys of protein/protein interactions and gene co-expression is a useful strategy to predict the functions of these proteins. We first implemented this strategy in the FIViz tool (11). We have extended this work, in collaboration with the IDG/Illuminating the Druggable Genome project, to develop a robust, user-friendly web-based computational framework to associate human proteins not yet manually curated in Reactome with Reactome pathways. Our framework, trained with a random forest of 106 protein or gene pairwise relationship features, infers functional involvement of proteins in individual pathways based on pathway enrichment analysis and fuzzy logic based simulation. We validated these inferences by mining PubMed abstracts, analyzing independent single cell RNA-seq data and manually curating a sample of the inferred ‘dark’ protein pathway assignments. This framework is implemented as a web application, the Reactome IDG portal, https://idg.reactome.org. It can be applied to any protein not annotated in Reactome to identify candidate pathways in which the protein may function. It can also be applied to annotated proteins to identify crosstalk between pathways and fill other annotation gaps. Pilot work suggests that this strategy can generate ‘guilt by association’ relationships between about half the ∼6300 ‘dark’ proteins and Reactome pathways (12,13).

## Annotating germline and somatic genetic variation

The number of variants of human protein coding genes, whether discovered in targeted searches for causes of disease or inferred from genomic sequencing, is large and rapidly growing. Catalogs like ClinVar (14) and COSMIC (15) classify variants by their likely effects on protein structure and function, association with known disease phenotypes, and evidence quality.

**Figure 1.**
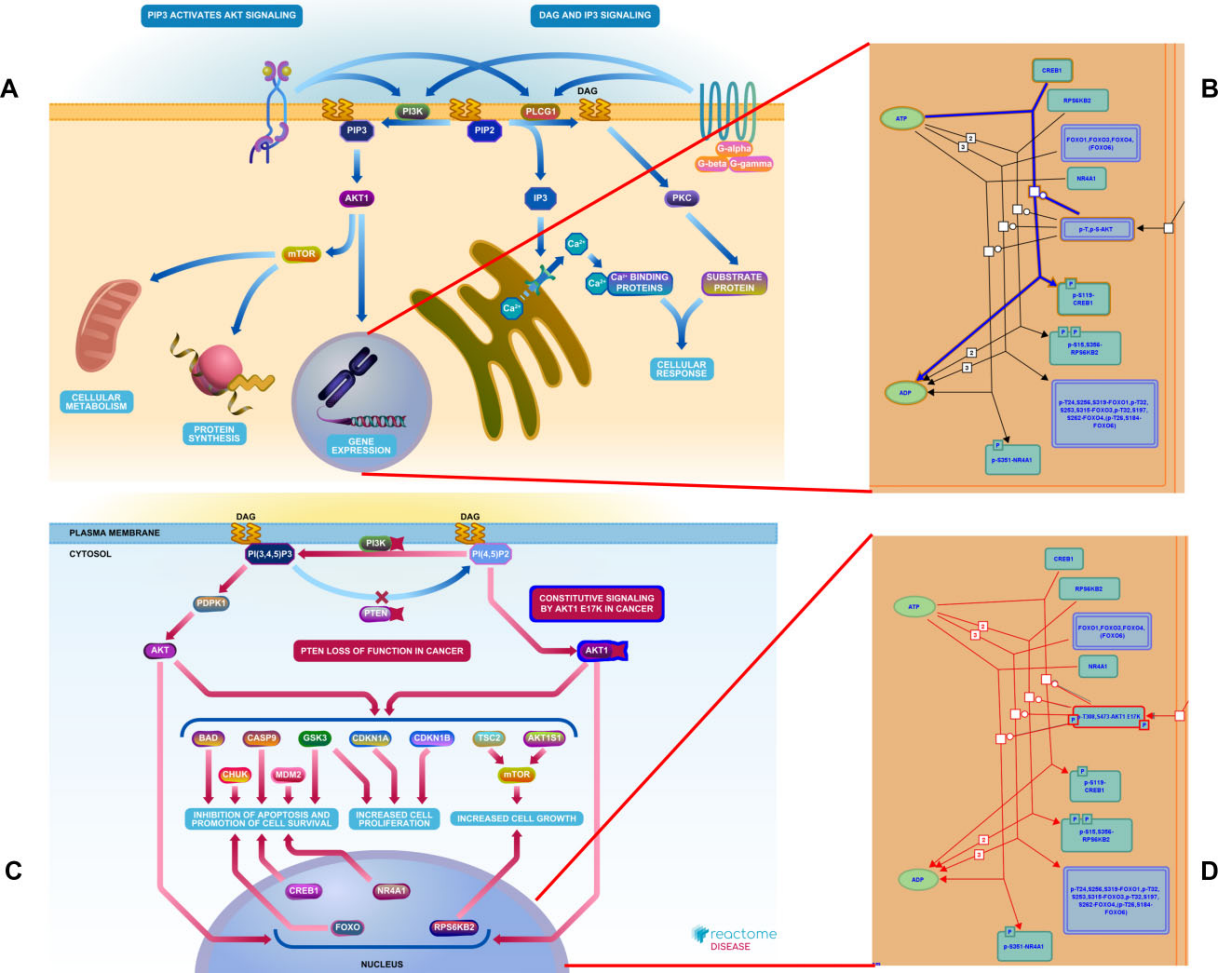
Disease variant-associated disease reactions in Reactome pathway diagrams. The pathway, ‘Intracellular signaling by second messengers (R-HSA-9006925)’ **(A)** includes a subpathway, ‘AKT phosphorylates targets in the nucleus (R-HSA-198693)’ **(B)** that shows normally regulated signaling mediated by AKT. The disease counterpart of the signaling pathway, ‘PI3K/AKT Signaling in Cancer (R-HSA-2219528) **(C)** includes a subpathway, ‘Constitutive Signaling by AKT1 E17K in Cancer (R-HSA-5674400)’ **(D)** that shows constitutive AKT signaling due to a missense mutation that enables AKT to bypass normal negative regulation and become constitutively active.

If a germline or somatic mutation leads to expression of a protein gene product that has lost its normal function or that has gained a novel one, annotation of the variant protein and of the resulting variant reaction in Reactome is straightforward in principle. Just as a co- or post-translational modification of a protein is annotated as replacement of a specified amino acid residue with a covalently modified one, germline and somatic genetic variants are annotated as the replacement of the amino acid residue normally found at a position by a different one. Our annotation strategy enables us to distinguish two types of reactions involving these variant proteins. If the variant protein has lost its normal function, reactions that require the wild-type protein as an input, catalyst, or regulator fail, having inputs but no outputs. If a variant gene product has gained a novel function or lost sensitivity to normal regulatory processes, reactions result with abnormal outputs or normal outputs under abnormal conditions (Figure 1).

Reactome thereby allows visualization of effects of mutations in a pathway context: what causally downstream processes might be expected to fail as the result of loss of function in a protein in the pathway? Do bypass processes exist that might relieve these effects? What abnormalities might be expected if a protein in a signaling process that is normally tightly regulated becomes constitutively active as the result of a gain of function mutation? Cross-referencing each variant reaction to its normal counterpart and thus to the pathway or pathways in which the normal counterpart functions allows the generation and side-by-side comparison of normal and variant/mutant reaction networks, enabling users to visualize possible disruptive effects of the mutated protein.

The Reactome curation process, however, cannot scale to encompass existing catalogs of pathogenic mutations and their continued rapid growth. Rather, our curation efforts have focussed on mining these catalogs to identify well-characterized ‘type’ variants, each associated with a qualitatively distinct molecular phenotype such as changed catalytic activity, insensitivity to a normal negative regulator, sensitivity to a drug that has no effect on its normal counterpart, or gain of resistance to a drug that inhibits other variants. Our annotation of the pathway ‘Signaling by ERBB2 in Cancer’ (R-HSA-1227990), for example, illustrates the range of mechanisms by which normal ERBB2 signaling is disrupted and the types of small-molecule drugs that target these ERBB2 variants. In this way, Reactome enables a clinical investigator with a novel variant that can be classified based on the appropriate catalog, or perhaps by a computational tool such as AlphaMissense (16), to use Reactome to form specific, testable hypotheses for its likely effects at a pathway level and its drug susceptibility.

## Drugs

In the same way that the Reactome data model allows the easy extension of annotation of covalent modifications of proteins to capture changes due to genetic variation, the model allows the effects of drugs, both small molecules and macromolecules such as RNAs and monoclonal antibodies, to be annotated in two-step reaction sequences. In the first, the drug binds its target human protein. In the second, the drug:protein complex regulates the reaction that the protein would otherwise mediate. Using this model, we have annotated, so far, the effects of 1033 chemical drugs and 86 protein drugs. As in the case of genetic variant proteins, the network organization of Reactome allows easy visualization of downstream effects of drugs. And also as in the case of genetic variants, scaling even this basic annotation process to encompass the vast and growing catalogs of drugs and their targets is impractical. To date, we have focused manual annotation on drugs and processes of immediate clinical interest, such as coronavirus infection (R-HSA-9679191) and fibrin clot formation in blood coagulation (R-HSA-140877). Also, data visualization features, such as automatic overlay of drug interactions on the top of pathway diagrams, have been implemented in the Reactome main web site and the Reactome IDG portal, assisting researchers to infer the potential impacts of drugs on pathways without manual curation.

Building on our annotations of drug interactions with their human protein targets, we have begun to annotate the entire ADME (absorption, distribution, metabolism, excretion) life cycle of selected drugs, e.g. ribavirin (R-HSA-9755088), in collaboration with PharmGKB (17), potentially facilitating the visualization of interactions and off-target effects of drugs of interest.

## Extensions of Reactome

### Molecular annotations above the single-cell level

The Reactome project, at its inception, was envisioned as building a comprehensive parts list of all reactions enabled by human proteins, onto which a user could overlay a list of proteins expressed in a tissue of interest under physiological conditions of interest to infer a tissue- and condition-specific reaction network (7,8). As discussed above, experimental data needed to construct a full list is lacking, and computational tools are insufficient to fill gaps and infer tissue or cell type specific reaction networks.

**Figure 2.**
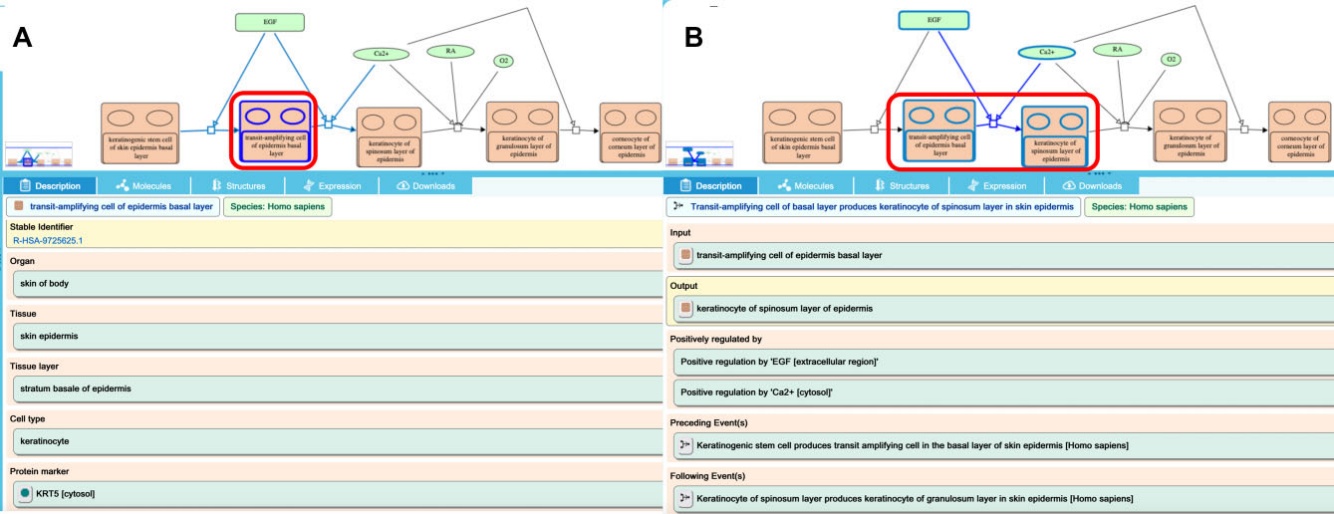
Annotation of cell- and tissue-specific reactions. A cell lineage path, ‘Development of keratinocytes in interfollicular skin epidermis’ (R-HSA-9725554) is represented as four cell development steps, equivalent to reactions. Each step transforms an input cell, equivalent to a physical entity, to an output cell, regulated by physical entities such as epidermal growth factor (EGF) and calcium ions (Ca^2+^). **(A)** A cell icon, ‘transit-amplifying cell of epidermis basal layer’ (R-HSA-9725625), highlighted with a red box in the pathway diagram panel (above) is annotated with cell and tissue ontology terms to identify and distinguish it from other kinds of cells and with protein and RNA markers that are key to its function in the process annotated here, listed in the details panel below the diagram. **(B)** A cell development step, ‘Transit-amplifying cell of basal layer produces keratinocyte of spinosum layer in skin epidermis’ (R-HSA-9727354) highlighted with a red box in the pathway diagram panel (above) is annotated with regulators of the transformation and identities of preceding and following development steps in the details panel below the diagram.

To work around this limitation we have extended the event class of the Reactome data model and added new visualization features in the Reactome web site to allow explicit annotation of cell- and tissue-specific reactions. Annotation has focused initially on differentiation processes. In the same way that molecular events capture the transformation of input physical entities into output ones by means of molecular functions such as catalysis, transport, and binding, cell development steps are grouped into cell lineage paths. A new entity type, cell, allows annotation of a cell type with terms from cell and tissue ontologies (Cell Ontology (18), and Uberon (19), respectively) and with associated proteins and RNAs as markers that identify and individuate the cell (Figure 2A). Development steps have cell types as inputs and outputs and are regulated by small molecules such as calcium ions and proteins such as growth factors (Figure 2B). These molecular attributes and their functions link cell- and tissue-level annotations of differentiation processes to the subcellular molecular processes already systematically annotated in Reactome.

### Releasing material with limited review

To accommodate difficulties in getting external reviews of new and revised material, we are allowing release of limited numbers of events without such review, tagged to indicate their potentially lower reliability. Material that has received full internal and external expert reviews (all released content before June 2023) has a 5-star review status rating. New material that has been curated and fully reviewed by a second curator but for which we have been unable to obtain an expert external review after six months is released with a 3-star rating. Previously fully-reviewed (5-star review status) events whose key attributes (participating entities for a reaction; participating reactions for a pathway) have been changed are re-released with a 4-star rating after independent internal review.

Three- and four-star events (https://reactome.org/userguide/review-status) were first released in June 2023 (V85). So far, this policy change has allowed the release of 122 finished events without external review (three-star), e.g. ‘NFE2L2 regulating MDR associated enzymes’, and of 12 previously released events that have been revised but not re-reviewed (four-star), e.g. ‘SUMOylation of immune response proteins’.

## Interoperability and resilience of data resources

Information resources like Reactome are most useful not in isolation but as parts of an integrated resource community. This kind of interoperability is a central feature of Reactome: reliable and widely-used reference resources provide the core information on which Reactome event annotations are assembled. For example, canonical forms of proteins are taken from UniProt (20), then annotated locally to describe changes from that canonical form, e.g. covalent or genetic modifications, and to add reaction-specific attributes such as subcellular localization. The controlled vocabulary of molecular functions, biological processes, and cellular components, and the logical relationships among these terms are taken from the Gene Ontology (9). Small molecules are from ChEBI (21) and the chemical equations for charge- and mass-balanced reactions occurring at physiological pH are from RHEA (1). As part of our own literature-based curation process, if we discover a discrepancy between information in one of these resources and new information we are annotating, we consult with the other resources to resolve the discrepancy. We also regularly check our annotations against these other resources to uncover and resolve discrepancies due to changes in the latter.

This approach to designing data resources has yielded a high level of interoperability among them. A user, viewing the UniProt entry for a protein or the ChEBI entry for a chemical, can navigate to the Reactome representations of reactions involving those entities. The first part of this dynamic, resource integration to promote interoperability, is codified in the FAIR (**F**indable, **A**ccessible, **I**nteroperable, **R**eusable - 22) and TRUST (**T**ransparency, **R**esponsibility, **U**ser community, **S**ustainability, **T**echnology - https://datascience.nih.gov/sites/default/files/NIH_Workshop_on_Trustworthy_Data_Repositories_Report_7-8-2019%20FINAL.pdf) principles, and entities like ELIXIR Core Data Resources (23) and the Global Core Biodata Coalition - https://globalbiodata.org/ promote it. Reactome is a part of the ELIXIR infrastructure and is a Global Core Data Resource.

Interoperability, however, creates a high level of interdependence: if one resource cannot maintain its current data and add new information as it accrues, or if in response to availability of new data types and user needs a resource changes the range (scope) or degree of specificity (granularity) of its annotations, the resulting gaps and discrepancies propagate to all of the resources. In the past two years, for example, 1990 (4.5%) of the 43300 terms in the Gene Ontology have been changed by additions, obsoletions, or merges (9). And UniProt, enforcing the standard that there should be one canonical protein entry for each gene product, has collapsed their previous collection of hundreds of HLA-A, B, C and D genes into four, each with an extraordinary number of polymorphic alleles on the basis of extensive resequencing of this region of the human genome (24). Management of such changes in all of the interdependent resources is essential to maintain both the quality of individual resources and their interoperability. But this requires continuing, skilled (expensive) manual work and can have unpredictable downstream effects on usage of annotated data for studies such as gene overexpression analyses. These are hard problems but collaborative work aimed at aligning Reactome process-description pathways content systematically with GO activity-flow models (25,26) and at coordinating annotation of Reactome human pathways with corresponding mouse ones to develop a resource to analyze disease phenotypes between the two species (27) promises to provide good solutions to them.

## Conclusions

The Reactome Knowledgebase of the molecular details of human biological processes continues to grow in size and scope. Since the last NAR update, Reactome has extended its annotations of genetic variation associated with disease, and of small-molecule drugs, including ones that target the protein products of genetic variants. Software developments include a protocol to expedite release of selected new and updated information without full external review, and a framework for cell type- and tissue type-specific annotations. Finally, we consider how to best maintain and improve Reactome's interoperability with other resources such as GO and UniProt in accord with FAIR and TRUST principles.

## Data Availability

Reactome is open-source and open-access. All Reactome data are available in various formats from our downloads page (https://reactome.org/download-data). A history tool is under development to enable users to track changes in our data over time. All software is available from our GitHub repositories (https://github.com/reactome and https://github.com/reactome-pwp), under terms that allow for free reuse and redistribution. We have created Zenodo packages for the versions of our software and data discussed in the article: Pathway Browser: 10.5281/zenodo.10022792 (Frontend for the PathwayBrowser) Data-content: 10.5281/zenodo.10022911 (Frontend for the search, detail, schema and icon library pages) CuratorTool: 10.5281/zenodo.10022856 (Local software to create reactome data) Content-Service: 10.5281/zenodo.10022866 (Backend API to access content of Reactome) Analysis-Service: 10.5281/zenodo.10022873 (Backend API to perform analysis on Reactome) Reactome Data 86: 10.5281/zenodo.10018440 (Data dump of version 86, both Neo4j and MySQL database)
